# Exploration of UK Lotto results classified into two periods

**DOI:** 10.1016/j.dib.2017.07.037

**Published:** 2017-07-20

**Authors:** Hilary I. Okagbue, Muminu O. Adamu, Pelumi E. Oguntunde, Abiodun A. Opanuga, Manoj K. Rastogi

**Affiliations:** a Department of Mathematics, Covenant University, Ota, Nigeria; b Department of Mathematics, University of Lagos, Akoka, Lagos, Nigeria; c National Institute of Pharmaceutical Education and Research, Hajipur 844102, India

**Keywords:** Digital root, Lottery, Lotto, Randomness, Statistics, United Kingdom

## Abstract

United Kingdom Lotto results are obtained from urn containing some numbers of which six winning numbers and one bonus are drawn at each draw event. There is always a need from prospective players for analysis that can aid them in increasing their chances of winning. In this paper, historical data of the United Kingdom Lotto results were grouped into two periods (19/11/1994–7/10/2015 and 10/10/2015–10/5/2017). The classification was as a result of increase of the lotto numbers from 49 to 59. Exploratory statistical and mathematical tools were used to obtain new patterns of winning numbers. The data can provide insights on the random nature and distribution of the winning numbers and help prospective players in increasing their chances of winning the lotto.

## Specification Table

**Table:** 

Subject area	Decision Science
More specific subject area	Lottery Statistics/Gambling Theory
Type of data	Table
How data was acquired	The data was retrieved from www.lottery.co.uk[1]
Data format	Processed data from November 19, 1994 to May 10, 2017
Experimental factors	Data refined from the results archived in www.lottery.co.uk, only the single cases were considered
Experimental features	Statistical analysis, digital root analysis.
Data source location	United Kingdom
Data accessibility	All the data are in this data article.
Software	Microsoft Excel and Minitab 17 Statistical Software

## Value of the data

● The data analysis provides a different approach of classifying winning numbers of the UK lotto results [1], [2], [3], [4], [5], [6].
● The data analysis can be extended to winning pairs and triples.
● The use of digital root provides another avenue for studying probabilities of winning [7], [8].
● Discovery of new patterns can encourage more players thereby improving the economic conditions and welfare of the country [9], [10], [11].
● The data can be useful for educational purposes and gambling researchers, number theorists, lotto operators, statisticians, journalists and so on.
● The method and analysis can be replicated for other lotto game results.

## 1 Data

The data for this study has been analysed to a certain extent, archived and updated at each draw in [1]. This data article contains data generated from different approach other than what was contained in [1] and it is publicly available. The data was on gathered on draw by draw basis. The data is divided into two periods; period A: when the lotto numbers are from one to forty nine (19/11/1994–7/10/2015) and period B: when the lotto numbers are from one to fifty nine (10/10/2015–10/5/2017). The draws for periods A and B are 2065 and 166 respectively. The data obtained for periods A and B when the winning numbers are classified using certain number criteria are shown in Table 1, Table 2, Table 3, Table 4. The frequency distribution of the lotto winning numbers when they are classified according to their digital roots is shown in Table 6 and the various lotto numbers that constitute each digital root are listed in Table 5. This article also introduces the use of the frequencies of digital root in chi-square tests. Finally, simulated data showed the uniformity, randomness and non-normality of occurrence of winning numbers in UK lotto game.

**Table 1:** The lotto single winning numbers classified in decimal (base 10).

**Numbers**	**Period A**	**Period B**
1–10	2488	157
11–20	2423	174
21–30	2577	165
31–40	2601	181
41–50	2301	157
51–59		162

The most single winning numbers from period A corresponds to 31–40 and the least corresponds to 41–50. Understandingly, the last class contains only 9 numbers for period A. Currently, from the analysis, prospective players with numbers 31–40 and 11–20 has more frequency than other classes.

**Table 2:** The lotto single winning numbers classified in multiples.

**Multiples**	**Period A**	**Period B**
2	6036	499
3	4093	294
4	2993	226
5	2293	167
6	2053	145
7	1761	130
8	1511	111
9	1277	94
10	1026	82

**Remark**: The frequency of occurrence decreases with increasing multiples of number for both periods.

**Table 3:** The lotto single winning numbers classified in odd and even numbers.

**Numbers**	**Period A**	**Period B**
Even	6036	499
Odd	6354	497
Total	12,390	996

**Remark:** More single odd winning numbers were drawn in period A. However, almost the same frequency was drawn for both even and odd single winning numbers in period B. Chi-square tests and t-tests may not be useful in confirmation the result since the possible winning numbers are more than the even numbers by one.

**Table 4:** The lotto single winning numbers classified in prime and non-prime numbers.

**Numbers**	**Period A**	**Period B**
Prime	3770	307
Non-prime	8620	689
Total	12,390	996

**Remark:** Prime numbers appeared in 27% and 31% of all the single winning numbers in periods A and B respectively.

**Table 5:** The lotto single winning numbers classified in digital roots 1–9.

**Digital root**	**Lotto numbers**
1	1 10 19 28 37 46 55
2	2 11 20 29 38 47 56
3	3 12 21 30 39 48 57
4	4 13 22 31 40 49 58
5	5 14 23 32 41 50 59
6	6 15 24 33 42 51
7	7 16 25 34 43 52
8	8 17 26 35 44 53
9	9 18 27 36 45 54

## 2 Methods and materials

Various aspects of statistical, mathematical and psychological analysis of lottery have been considered [12], [13], [14], [15], [16], [17], [18].

### 2.1 Digital root

This is the sum of digits of a studied number until a single digit number is the final outcome [19], [20], [21], [22]. Digital roots often reveal hidden patterns of distributions as seen in [23], [24], [25]. This can be applied to lotto to reveal hidden patterns of distribution of winning numbers. The complete list of numbers grouped under their respective digital roots and is shown in Table 5. The digital root of the single winning numbers for periods A and B is shown in Table 6.

**Table 6:** The frequency distribution of the single winning numbers classified according to their digital root for periods A and B.

**Digital root**	**Period A**	**Period B**
1	1467	127
2	1513	128
3	1532	113
4	1526	126
5	1254	119
6	1284	87
7	1258	107
8	1279	94
9	1277	82

**Remark:** The importance of the digital roots is that it makes use of all the observations unlike what was obtained in Table 2, where the multiples excluded the prime numbers.

### 2.2 Chi-square test of independence

The Pearson chi-square test is conducted to determine whether the observed values conform to theoretical expectations. The expected frequencies in the chi-square test of independence follow the uniform distribution. Details on chi-square test and other tests can be found in [26], [27], [28], [29], [30], [31]. This paper introduces the use of frequency obtained from the digital roots of number instead of all the numbers in chi-square test of independence. This approach was compared with the traditional procedure using the frequency data in totality. The results of the Chi-square tests for periods A and B using Table 6 are shown in Table 7, Table 9 while the decision rule based on different confidence intervals are shown in Table 8, Table 10.

**Table 7:** The chi-square test for period A.

**Number**	**Observed**	**Expected**	**Residual**	**Statistic**
1	1467	1517.22	−50.22	1.662282596
2	1513	1517.22	−4.22	0.01173752
3	1532	1517.22	14.78	0.143979383
4	1526	1517.22	8.78	0.05080898
5	1254	1264.22	−10.22	0.082618848
6	1284	1264.22	19.78	0.309478097
7	1258	1264.22	−6.22	0.030602585
8	1279	1264.22	14.78	0.172793027
9	1277	1264.22	12.78	0.12919302
				2.593494056

**Table 8:** Decision rule for the chi-square test for period A.

**Α**	**0.995**	**0.99**	**0.975**	**0.95**	**0.90**	**0.10**	**0.05**	**0.025**	**0.010**
**Decision**	Reject H_0_	Reject H_0_	Reject H_0_	Accept H_0_	Accept H_0_	Accept H_0_	Accept H_0_	Accept H_0_	Accept H_0_

*Note:* α denotes the level of significance

**Table 9:** The chi-square test for period B.

**Number**	**Observed**	**Expected**	**Residual**	**Statistic**
1	127	118.17	8.83	0.659802826
2	128	118.17	9.83	0.817710925
3	113	118.17	−5.17	0.226190234
4	126	118.17	7.83	0.518819497
5	119	118.17	0.83	0.005829737
6	87	101.28	−14.28	2.013412322
7	107	101.28	5.72	0.323048973
8	94	101.28	−7.28	0.52328594
9	82	101.28	−19.28	3.670205371
				8.758306

**Table 10:** Decision rule for the chi-square test for period B.

**Α**	**0.995**	**0.99**	**0.975**	**0.95**	**0.90**	**0.10**	**0.05**	**0.025**	**0.010**
**Decision**	Reject H_0_	Reject H_0_	Reject H_0_	Reject H_0_	Reject H_0_	Accept H_0_	Accept H_0_	Accept H_0_	Accept H_0_

*Note:* H_0_ denotes the null hypothesis (observations are random)

The expected value was obtained from Table 6 by the sum of all the values under the column (Period A) divided by 9.

The statistical hypothesis is stated; null hypothesis imply independence while the alternative imply otherwise.

$\chi_{cal}<\chi_{sig}$ Accept the null hypothesis (independence);

$\chi_{cal}>\chi_{sig}$ Accept the alternative hypothesis (association);

$\chi_{cal}=\begin{array}{c} 2.593494056 \end{array}$ (From Table 7).

The decision rule for the different level of significance of the chi-square test for period A is shown in Table 8.

The expected value was obtained from Table 6 by the sum of all the values under the column (Period B) divided by 9.

The statistical hypothesis is stated; null hypothesis imply independence while the alternative imply otherwise.

$\chi_{cal}<\chi_{sig}$ Accept the null hypothesis (independence);

$\chi_{cal}>\chi_{sig}$ Accept the alternative hypothesis (association);

$\chi_{cal}=\begin{array}{c} 8.758306 \end{array}$ (From Table 9).

The decision rule for the different level of significance of the chi-square test for period B is shown in Table 10.

The basis for the statistical decision is that the calculated chi-square statistic is compared with the one tabulated at different degrees of freedom. This revealed that the distribution of the winning numbers of UK lotto is purely random especially at high confidence intervals. This has shown that the UK lotto game is fair.

### 2.3 Simulation analysis

**Fig. 1:**
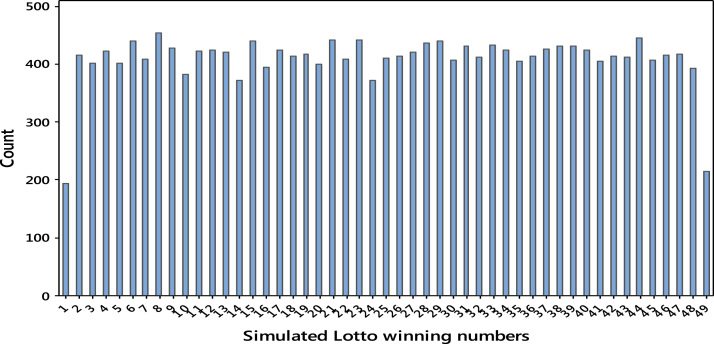
Simulation results for Period A.

**Fig. 2:**
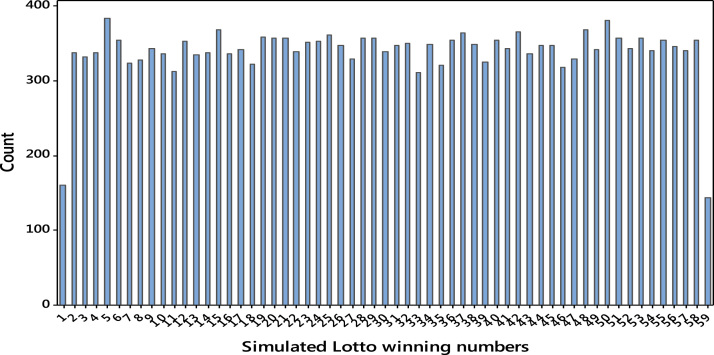
Simulation results for Period B.

Monte Carlo simulation was used to generate 20,000 simulated results using the discrete uniform distributions for periods A and B. The results are shown as histograms in Fig. 1, Fig. 2.

The simulation results revealed the uniformity in frequency distributions of the lotto numbers and hence the winning numbers does not appear to cluster around any specific value. However, the extreme values 1, 49 and 59 seem to deviate from uniformity. This is one of the major drawbacks of Monte Carlo simulation used to generate those results.
